# Sustainable Management of Food Waste during COVID-19 Pandemic: Insights into Irrational Food Hoarding among Chinese Citizens

**DOI:** 10.3390/foods11244049

**Published:** 2022-12-14

**Authors:** Kangjie Zhang, Fuduo Li, Huanli Li, Changbin Yin

**Affiliations:** 1 Rural Development Institute, Chinese Academy of Social Sciences, Beijing 100732, China; 2 Institute of Agricultural Resources and Regional Planning, Chinese Academy of Agricultural Sciences, Beijing 100081, China; 3 College of Economics and Management, Northwest Agriculture and Forestry University, Yangling, Xianyang 712100, China; 4 Research Center for Agricultural Green Development in China, Beijing 100081, China

**Keywords:** food waste, irrational food hoarding behavior, hoarding preference, psychological panic, group influence

## Abstract

During the COVID-19 pandemic, food waste caused by excessive hoarding has accounted a large proportion of the total food waste in urban Chinese households, which indicates that reducing food hoarding has become key to managing household food waste. This study therefore explored the behavioral mechanisms underlying excessive food hoarding among citizens. Based on a sample set of 511 respondents surveyed in Beijing, Hefei, and Guiyang in July 2022, a PLS-SEM model was conducted using SmartPLS 3.0 software to simulate the decision-making process of food hoarding. The following results were found. First, among the households with hoarding, 66.37% had some degree of food waste. Second, hoarding preference was the direct predictor of hoarding behavior, which means that hoarding behavior can be effectively controlled by regulating preferences. Third, group influence including homology consistency and social network support, as well as psychological panic, both enhanced citizens’ hoarding preference and induced hoarding behavior. Therefore, it is necessary to weaken group influence and try to help citizens overcome panic. Finally, food supply information release can not only alleviate citizens’ psychological panic and weaken group influence, but also block the transformation of preference into behavior. The above results are of great importance for the design of management policies for food waste caused by irrational hoarding during the pandemic.

## 1. Introduction

At present, food waste has become one of the major threats to global food security. According to estimates by the FAO, 13 billion tons of food is wasted globally each year, accounting for 32% of total annual food production. Food waste is also a major problem in China. In total, between 17 and 18 million tons of food is wasted by Chinese families every year, which is enough to feed 30 to 50 million people [1]. In addition to raising food prices and exacerbating food supply crises, food waste also has a negative impact on the environment, as it causes unnecessary greenhouse gas emissions and the inefficient use of water and land resources [2,3]. As agricultural resources and environmental constraints increase, reducing food waste has become a pivotal solution for sustainable development of the food system in China and even the world.

Food waste from households accounts for the majority of total waste along the food supply chain, both in advanced economies such as the United States and the European Union and in developing economies such as China and India [4]. There are two main sources of household food waste. The first is “waste at the table”, that is, waste that is produced when cooked food is not completely eaten [5]. Another is “waste in storage cabinets”, that is, waste that is produced due to excessive hoarding [6]. During the COVID-19 pandemic, food hoarding became an important behavior for citizens trying to cope with external risks. Since most food, especially fresh food, is not resistant to storage, excessive hoarding will inevitably cause part of the food to rot, resulting in a large amount of food waste [7]. According to a survey, food waste caused by hoarding accounted for 65% of total food waste in urban households in China. Therefore, it is urgent to manage citizens’ irrational food hoarding when seeking to reduce household food waste.

Revealing the formation mechanism of citizens’ irrational food hoarding is the premise of behavioral regulation. Traditional behavioral economic theories suggest that demographic factors [8,9], cognitive factors [10,11], and various factors related to cost–benefit trade-offs [12,13] jointly determine individual behaviors. However, these factors have weak explanatory power for citizens’ irrational food hoarding behavior (IFHB) because they do not take into account the impact of specific situational factors. Forbes et al. [14] and Taylor [15] both suggested that external stimuli, especially sudden stimuli, could induce psychological shock in citizens, which could lead to citizens making irrational coping decisions. Owing to the pandemic, people are at greater risk of food shortages and may suffer from constant psychological panic (PPA). To cope with potential risks, people are more inclined to hoard food in their daily lives.

Due to the limited ability of individuals to process information related to the impacts of COVID-19 on food supply, group influence emerges as an important driver of decision making among citizens. Groups are characterized by irrationality and impulsivity [16]. Under the influence of the group, individual behavior is almost on the verge of losing control [17]. Homology consistency (HCO) and social network support (SNS) are two main aspects of group influence. HCO means that being consistent with a group makes it easy to obtain evidence of the correctness of one’s own decisions [18]; SNS means that being in line with a group makes it easier to gain people’s recognition or admiration [19]. When individuals are exposed to the risk of outbreaks, they are completely unsure whether their behavior is correct. Under the influence of HCO and SNS effects, citizens are prone to use the behavior of others to guide their own actions, and thus are more likely to make irrational food hoarding decisions.

In addition, asymmetric market information often leads individuals to make irrational decisions [20]. This conclusion has been confirmed by previous studies. For instance, Kaneko et al. [21] demonstrated that incomplete information will not only lead to low market efficiency, but also induce consumers to make irrational purchase decisions. Noah et al. [22] reported that, in the context of incomplete information, it is difficult for citizens to make the decision to recycle food waste. In our case, consumers did not have access to sufficient market information, which prevented them from adjusting their food hoarding behavior. Under these circumstances, government food supply information release (FSIR) eliminates the information asymmetry faced by citizens in the food market [23], thus emerging as a potential means to regulate citizens’ IFHB and reduce food waste.

Therefore, the overall goal of this study is to develop an optimization framework suitable for the COVID-19 pandemic scenario which can fully reveal the behavioral mechanism of irrational food hoarding among Chinese citizens, thus helping to better realize the sustainable management of food waste. Specifically, this paper aims to address the following: (1) how to integrate PPA, HCO, SNS, and FSIR to construct an analytical framework with strong explanatory power and broad application prospects, (2) how to determine influence paths of the above components on IFHB, and (3) how to assess the extent of such influences.

This paper contributes to the existing literature in two aspects. On the one hand, this study focuses on food waste caused by irrational food hoarding during the COVID-19 pandemic. Although previous studies have explored the issue of household food waste, almost all of these studies focused on “waste at the table” and less on “waste in storage cabinets”. Our exploration is probably one of the more cutting-edge ones, and the expected findings can be provided to policy makers to offer insight into the development of comprehensive food waste management schemes. On the other hand, the analytical framework constructed in this study breaks through the limitations of traditional behavioral analysis frameworks and innovatively introduces indicators that can better reflect the psychological changes citizens undergo during a pandemic to reveal their behavioral responses. This novel framework is well embedded with situational factors, which makes it more explanatory.

The rest of the study is organized into five parts: the second part introduces the research background and the construction process for the analytical framework and puts forward the research hypothesis; the third part introduces the materials and methods, including the questionnaire design, descriptive statistical analysis of the collected data, and the research methods used in this paper; the fourth part contains the empirical analysis results; the fifth part is the discussion of the results; and conclusions and limitations are arranged in the sixth part.

## 2. Research Background and Theoretical Framework

### 2.1. Research Background: Food Waste Caused by IFHB during the COVID-19 Pandemic

In this study, hoarding behavior is not “pathological collecting” as defined in medicine, but rather the coping actions taken by citizens to improve their psychological security in response to the potential threat of the COVID-19 pandemic to their supply of food. Before the outbreak of COVID-19, the incidence rate of irrational food hoarding among the population was low, only about 2.3–5.8%; however, after the outbreak, this rate increased dramatically [1]. More importantly, there appears to be a trend of people hoarding food that emerged amid the pandemic, and that trend is not going to reverse anytime soon [24].

**Figure 1:**
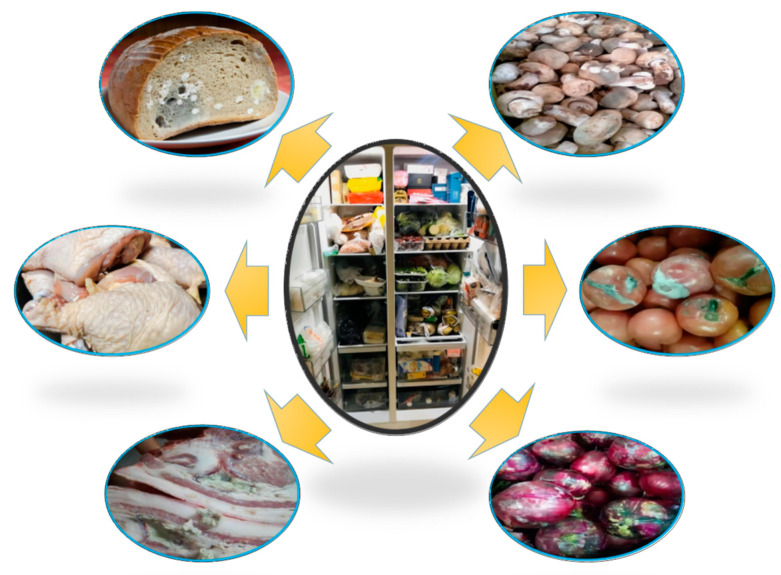
Food waste caused by IFHB.

Irrational food hoarding causes large amounts of food waste (Figure 1). This is because, on the one hand, the amount of food stored greatly exceeds a household’s capacity to consume that food within a certain period of time, and the remaining food is no longer suitable to eat because it has already decomposed [25]. On the other hand, irrational food hoarding is catalyzed by “impulse” emotions, in which people do not just buy what their family prefers, but also gather whatever they can for a rainy day. In this situation, many foods that are not on a family’s preference list end up languishing in lockers and eventually rotting away. A recent survey showed that the proportion of food waste caused by irrational hoarding in Chinese urban households has risen from less than 10% before the outbreak to about 60% now [1]. Therefore, it is very important to pay attention to IFHB and food waste produced by citizens when aiming to improve public governance in the food economy.

### 2.2. Theoretical Framework: S-O-R Framework and Its Extension

**Figure 2:**
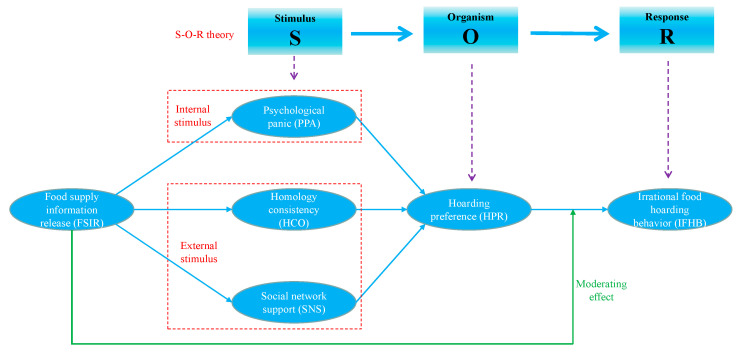
The research framework of this study.

The Stimulus(S)-Organism(O)-Response(R) model proposed by Mehrabian and Russell (1974) was chosen to construct the analytical framework of this study. Stimulus (S) refers to the internal and external environmental factors faced by the organism, organism (O) refers to a psychological transformation mechanism by which individuals internalize stimulus factors into information, and response (R) represents the relevant coping behaviors of individuals to the stimulus information [26,27]. PPA, HCO, and SNS were selected as stimulus factors, in which PPA was the internal stimulus and HCO and SNS were the external stimuli. Hoarding preference (HPR), a variable reflecting individual psychological changes, was selected as an organism factor, while IFHB, which we focused on, was selected as a behavioral response factor. To further improve the explanatory power of the model, FSIR was introduced as a moderating factor to extend the S-O-R framework. The integrated research framework is shown in Figure 2.

### 2.3. Research Hypothesis

#### 2.3.1. The Influence of PPA on HPR

Although the fatality rate of COVID-19 has been greatly reduced, new mutant strains continue to threaten humans [28]. In the face of potential risks, people’s PPA has never disappeared [29]. In addition to the immediate threat to people’s lives, the COVID-19 pandemic has also had a significant negative impact on people’s livelihoods [30,31]. Most notably, current prevention and control measures, such as regional closures and home isolation, have decreased the availability of food by blocking or interfering with the food supply chain. Food is the most important material for human beings, and its availability and abundance are directly related to people’s survival and quality of life [32]. Threats to the food supply are bound to further stoke panic among citizens. According to Slovic et al. [33], the generation of panic is highly related to the perceived degree of uncontrollable events and the lethality of consequences; the deeper the panic, the more likely it is to stimulate individuals into produce coping preferences or intentions—in our case, HPR. Accordingly, the following hypothesis was proposed:

**Hypothesis** **1** **(H1).**  *PPA induced citizens’ HPR during the COVID-19 pandemic.*

#### 2.3.2. The Influence of HCO and SNS on HPR

Group influence, similar to herd mentality, refers to the fact that individuals are affected by the behavior of a crowd and show consistency with the public in terms of their perception, judgment, and understanding [34]. In the psychological masterpiece “*The Crowd: A Study of the Popular Mind*”, Gustave [35] suggested that “the group is irrational and has extremely strong impulsivity, which makes the group return to the original nature of human beings and denies any obstacles between its own desire and the realization of the desire”. Under the influence of groups, individuals are more likely to exhibit irrational decision-making. HCO and SNS are two effects of group influence. The essence of the HCO effect is that when the situation is uncertain, the behavior of others is most informative. The nature of the effect of SNS indicates that being in line with the group can make people more acceptable [18,19]. During the COVID-19 pandemic, people could not sufficiently acquire information related to whether the outbreak would affect the food supply chain and the HCO effect and SNS effect often made individuals lose the ability to control their own consciousness, thus leading to their behavioral preferences becoming consistent with those of the public. Therefore, the following hypothesis was proposed:

**Hypothesis** **2** **(H2).**  *HCO and SNS stimulated citizens’ HPR during the COVID-19 pandemic.*

#### 2.3.3. The Relationship between HPR and IFHB

In the consumer economy, preference is defined as the degree to which a consumer likes a commodity or a combination of commodities [36,37], which can be identified and judged by objective indicators or psychological feelings [38]. Obviously, preference is essentially an emotion or a tendency [39]. In our case, a resident’s food hoarding preference can be defined as an underlying passion for hoarding food. According to the theory of behavioral preference, preference is a key factor that induces behavior [40]. When preferences are reinforced by external sudden events, the probability of their transformation into behavior will be greatly increased [41]. Throughout the COVID-19 pandemic, the food supply chain has been constantly under threat. No matter whether it is driven by rational or emotional factors, citizens’ food hoarding preference will likely be further enhanced, which will inevitably lead to the emergence of further food hoarding behavior. Thereby, the following hypothesis could be proposed:

**Hypothesis** **3** **(H3).**  *There was a positive correlation between HPR and IFHB.*

#### 2.3.4. The Influence of FSIR on the S-O-R Framework

Crisis communication theory demonstrates that crisis information release plays an indispensable and decisive role in public crisis event processing. Crisis information release refers to governments sharing crisis information with the public through timely releases when a public crisis occurs, which hopes to achieve the goals of suppressing public panic and enhancing public satisfaction in the government [42]. According to the theory, during the COVID-19 pandemic, only through timely FSIR and continuous improvements in information accuracy that protect citizens’ right to know can the government reduce the public’s PPA. Moreover, the improvement in public information literacy can also restrain their inner irrational “impulse”, thereby weakening the negative effect of group influence caused by HCO and SNS [43].

The influence of FSIR on the S-O-R framework is not only reflected by its effect on PPA, HCO, and SNS, but also in its moderation of the conversion of HPR to IFHB. Previous studies have shown that information release can regulate organisms into exhibiting specific behavioral responses, and those responses can be either positive or negative. For example, Li et al. [44] pointed out that information release promotes the transformation of consumers’ pro-environmental food purchase preference into actual purchase behavior, while Streletskaya et al. [45] and Wilson et al. [46] believe that information release hinders the conversion of consumers’ high willingness to pay for emerging food to actual payment behavior by eliminating cognitive bias. In our case, the food supply information released by the government is often positive, which helps to improve citizens’ sense of security and thus prevents the part of food hoarding preference that arises from an “impulse” from being converted into actual hoarding behavior. Based on the above analysis, we put forward the following hypotheses:

**Hypothesis** **4** **(H4).**  *FSIR had negative effects on PPA, HCO, and SNS.*

**Hypothesis** **5** **(H5).**  *FSIR could inhibit the conversion of HPR to IFHB.*

## 3. Material and Methodology

### 3.1. Survey Design

#### 3.1.1. Questionnaire and Pilot

We developed a questionnaire according to the components of the research framework (Figure 2) to collect data. The questionnaire consists of four parts. The first part focuses on COVID-19 exposure and includes the occurrence of the epidemic in the county/township/community, awareness of the epidemic, panic under the epidemic, and access to epidemic-related information. The second part focuses on food hoarding during the COVID-19 pandemic and mainly investigates citizens’ perception of food supply risk and food availability under the epidemic and the impact of group influence and government food supply information release on citizens’ food hoarding. The third part investigates food waste produced by food hoarding, while the final section examines the demographic characteristics of the respondents. It should be noted that only part of the information collected by the questionnaire has been used in this study, and other information will be used in another study.

After completing the preliminary design of the questionnaire, an online pilot survey was conducted in Beijing in May 2022, and 137 responses were obtained. The pilot data were used to remove unreliable items from the questionnaire and to refine the presentation of each question so as to improve its comprehensibility. After that, we obtained the official version of the questionnaire.

#### 3.1.2. Survey

The survey was conducted online. In early June 2022, the official version of the questionnaire (after revisions stemming from the pilot data) was submitted to “Questionnaire Star”, a professional online survey platform with a database of potential respondents which is operated by a survey company, Changsha Ranxing Information Technology Co., LTD. in Changsha city, Hunan province, China. In 2022, the platform had no less than 6.5 million customers in mainland China. To ensure the quality of data, the screening question related to the respondent’s residence was strictly set. In addition, to motivate respondents to be patient enough to answer each question carefully, the platform’s financial reward service was enabled. Furthermore, as part of the ethics statement, each questionnaire includes a contact e-mail for the investigator so that any questions arising during the response process can be addressed in a timely manner. The principles proposed by the National Oceanic and Atmospheric Administration (NOAA) were used to improve the validity and reliability of the online survey data. These principles state that researchers should (1) declare that the questionnaires are only used for scientific research without involving personal information disclosure; (2) reasonably lay out the questionnaire’s structure to ensure that each interview can be completed within 10 min; and (3) consider respondents to be inattentive, and thus excluded from the sample set, if they spend less than 20% of the average time on the survey.

A total of 536 responses were obtained in this study, among which 25 questionnaires were judged to be invalid; thus, 511 valid questionnaires were finally included, with 177 from Beijing, 170 from Hefei, and 164 from Guiyang. The reason why we chose these three cities to carry out the questionnaire survey is mainly based on the consideration of sample representativeness. Beijing, Hefei, and Guiyang are located in the east, middle, and west of China, respectively. It is well known that different regions in China have different economic levels and living habits with regard to residents, and the samples obtained from these three cities should be nationally representative.

#### 3.1.3. Reasonableness of Sample Size

The following formula was used to calculate the minimum sample size [47]:

(1) $$n=\frac{N}{1+N{\left(e\right)}^{2}}$$

where *N* is the population size of the study region (unit: ten thousand), *e* represents the precision level (5%), and *n* denotes the minimum sample size. According to China’s seventh National Census, the number of citizens in Beijing, Hefei, and Guiyang is 19.16, 5.68, and 4.79 million, respectively.

(2) $$n=\frac{2963}{1+2963\times 0.05^{2}}=353$$

Therefore, the sample size satisfies the constraint condition regarding minimum sample size.

### 3.2. Descriptive Statistical Analysis

Descriptive statistics of variables used as controls are presented in Table 1. Male respondents made up the majority of the total sample. Respondents aged 40 years old and under accounted for 74.75%, while those aged over 50 accounted for less than 8%. Regarding education, more than half of the respondents had professional training or a bachelor’s degree. More surprisingly, more than 40% had a master’s or doctoral degree. Of the total respondents, nearly 67% were married. More than half of the families had 3–4 members, and families with less than 3 members accounted for 27.98%. Regarding income, 62.62% of respondents had a monthly household income of less than CNY 20,000, among which a considerable number even had a monthly household income of less than CNY 10,000. The sample is roughly representative of Chinese citizens in terms of household size and monthly household income yet leans toward a slightly younger and higher educated demographic, which is common in online-based surveys.

### 3.3. Methodology

PLS-SEM was adopted to test for significant relationships between variables. PLS-SEM is an integrated model that consists of a measurement equation reflecting the relationship between latent variables and observed variables and a structural equation exhibiting the relationship between exogenous and endogenous latent variables (Li et al., 2021). Compared to traditional SEM, PLS-SEM is more applicable when the theoretical model is exploratory or expansive [48].

Structural model:

(3) η=βη+Γξ+ζ

where $\eta ,\beta ,\xi ,\zeta \in R^{n}$, $\Gamma \in R^{n\times n}$; η is an m×1 vector consisting of *m* endogenous latent variables; ξ is an n×1 vector consisting of *n* exogenous latent variables; and Γ is a matrix of coefficients with m×n structure, which exhibits the impact of the exogenous latent variable ξ on the endogenous latent variable η.

Since PLS represents a recursive relationship, therefore

(4) $$\eta_{j}=\sum_{i}\beta_{ji}\eta_{i}+\sum_{j}\gamma_{jb}\xi_{b}+\zeta_{j}$$

where $\beta_{ji}$ represents the coefficient of endogenous variables, $\gamma_{jb}$ is the coefficient of exogenous latent variables, and $\zeta_{j}$ is the endogenous residual variable.

Measurement model:

(5) $$X=\Lambda_{\xi }+\varepsilon_{x}$$

(6) $$Y=\Lambda_{\eta }+\varepsilon_{y}$$

where x,y indicate the observed variables of exogenous and endogenous latent variables ξ and η, respectively;$\Lambda_{\xi }$ is a matrix of coefficients with q×n structure; and $\Lambda_{\eta }$ is a matrix of coefficients with p×m structure.

SmartPLS 3.0 and Stata 16 were used to perform empirical analyses. Structural modeling was completed using SmartPLS 3.0 software, and the regression analysis was conducted in Stata 16.

## 4. Results

### 4.1. Less Hoarding, Less Waste: Food Waste Stemming from Hoarding

**Figure 3:**
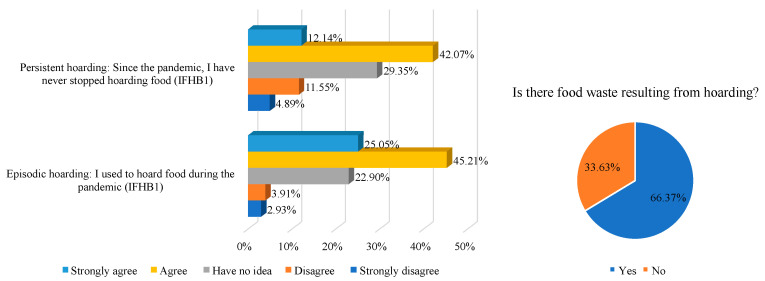
Citizens’ food hoarding behavior and food waste resulting from hoarding. Note: both episodic and persistent hoarding is considered to be food hoarding behavior.

In our survey, food hoarding was classified into episodic hoarding and persistent hoarding based on its duration, with these two variables measured by “I used to hoard food during the pandemic” and “Since the pandemic, I have not stopped food hoarding”, respectively. If the respondents agreed with these statements, they were judged to have the specific food hoarding behavior. Figure 3 shows that 70.26% and 54.21% of the residents agreed with the above statements, respectively. Among the households with hoarding behavior, 66.37% had some degree of food waste. It can be seen that, during the COVID-19 pandemic, food waste caused by hoarding behavior has been very serious indeed.

**Figure 4:**
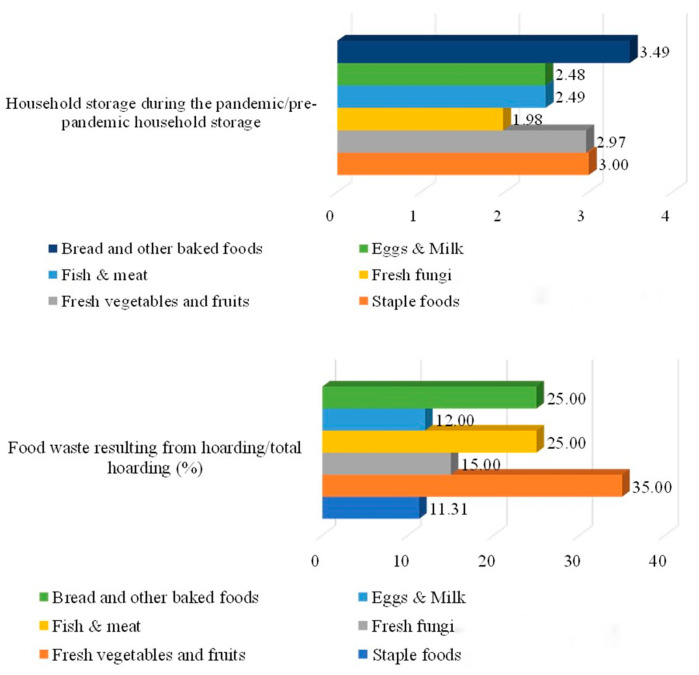
The structure of food hoarding and food waste.

Figure 4 demonstrates the structure of food hoarding and food waste. Compared to the pre-pandemic period, food hoarding by Chinese citizens has shown a marked increase. Among them, the hoarding of bread and other baked foods was the largest, which reached 3.49 times the household standing amount before the epidemic. This was followed by staple foods and fresh vegetables and fruits, both of which were about three times as much as before. The increase in household hoarding of eggs, milk, fish, and meat was also relatively large, reaching about 2.5 times the previous level. In addition, the hoarding of fresh fungi nearly doubled compared to before the pandemic. Of all the food that was being hoarded, 35% of fresh vegetables and fruits were wasted, as were 25% of fish, meat, bread, and other baked foods; the wasting of fresh fungi and eggs and milk due to spoilage accounted for 15% and 12% of their respective hoarding amounts.

The main causes of food waste produced by hoarding include (1) households storing much more food, which under no circumstances can they eat all of before some of it goes bad; and (2) the fact that food hoarded by citizens is not exactly what they really need, which will inevitably lead to some food being shelved for a long time before finally rotting away. There is an irrational element to the food hoarding of citizens during the pandemic. These “irrational” factors play an important role in inducing food waste. From this perspective, reducing the “irrational” component in household decision making is crucial to reducing food waste.

### 4.2. Citizens’ Behavioral Mechanisms of Hoarding Food

According to the above analysis, uncovering irrational food hoarding behavior among citizens resulting from the COVID-19 pandemic is an important prerequisite for combating food waste and can provide many valuable insights for policy makers. Therefore, this part explores the internal causes of citizens’ irrational food hoarding through structural modeling.

#### 4.2.1. Respondents’ Responses

PPA, HCO, SNS, HPR, and FSIR are the direct and indirect factors that affect citizens’ IFHB. An overview of the above latent variables and how they are measured is shown in Table 2. All measurement indicators were taken from existing studies and have been well validated. Minor corrections were made to wording to ensure that the items were compatible with the real context of this study.

**Figure 5:**
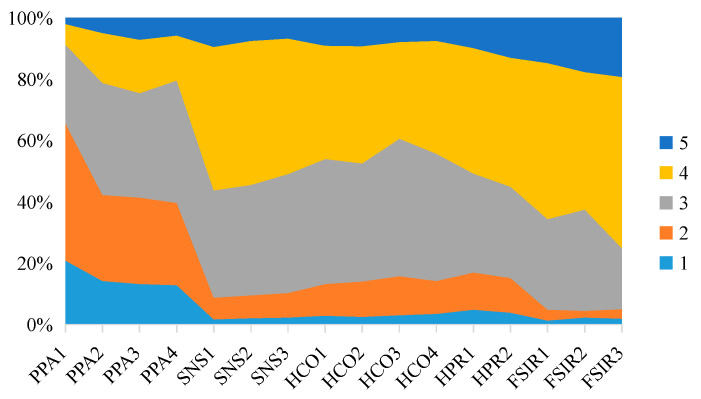
Descriptive statistics of respondents’ responses to survey items.

Figure 5 presents citizens’ responses to the survey items. A 5-point Likert scale ranging from “strongly disagree (1)” to “strongly agree (5)” was used for variable measures. A higher score indicates a higher level of agreement or conformity. It is obvious that only a small number of respondents expressed a relatively strong panic about the pandemic. For group influence, more than 50% of respondents explicitly said they would hoard food if people around them advised them to do so; approximately 40% of respondents reported that if they saw others hoarding food, they would take it for granted that they should do the same. A total of 50.68% of the surveyed citizens said they preferred to hoard food more now than before the pandemic, and 54.77% indicated that they will keep hoarding as long as the pandemic does not end. According to our survey, more than 60% of the respondents gave high ratings to the government’s efforts to release food supply information.

#### 4.2.2. Complete Random Analysis of Variance

**Figure 6:**
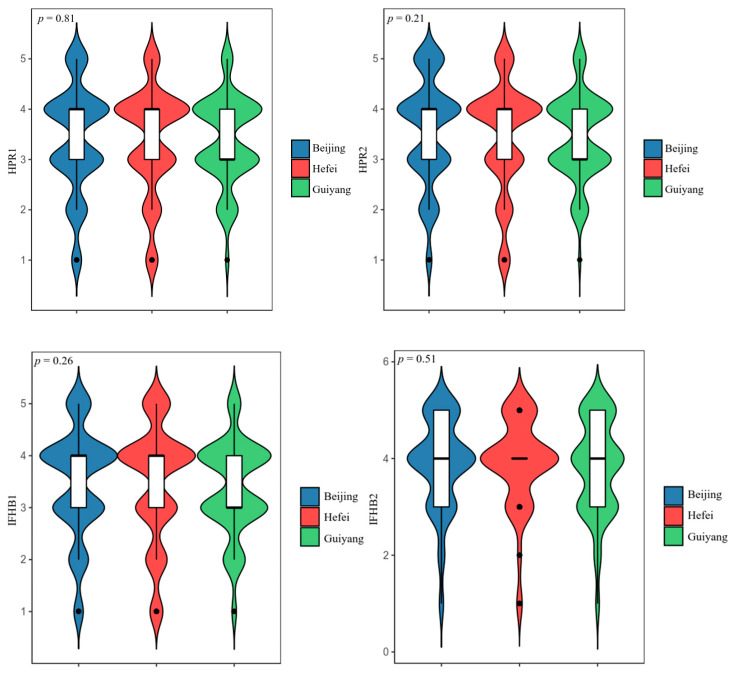
Heterogeneity test of HPR and IFHB among cities.

Considering that the research data were collected from three different cities in eastern, central, and western China, we sought to determine whether there were significant differences in residents’ food hoarding preferences and hoarding behaviors among these cities. Figure 6 shows that the *p*-values of the heterogeneity test of the two components of HPR are 0.81 and 0.21, and the *p*-values of the component heterogeneity test of IFHB are 0.26 and 0.51. Obviously, there is no significant difference in HPR and IFHB among different cities. This means that it is appropriate to use a full sample rather than a sub-sample for analysis in the empirical research that follows.

#### 4.2.3. Measurement Model

##### Reliability Testing

Reliability refers to the stability of the measurement results, and high reliability indicates a small measurement error [55]. Cronbach’s α and composite reliability (CR) are two commonly used metrics to evaluate reliability. Generally speaking, 0.7 is identified as the threshold limit of these two indicators [56]. In other words, the measurement model is considered reliable when the values of these two indicators are greater than 0.7 [54]. According to Table 3, all the indexes meet the corresponding constraint conditions in this study.

##### Validity Testing

Validity includes convergent validity and discriminant validity [57]. The most commonly used indicators to test convergent validity are average variance extracted (AVE) and factor loading. When AVE and factor loading are greater than 0.5, the measurement model can be deemed to have excellent convergent validity [54]. Of course, the higher the value, the higher the convergent validity will be. In our study, the values of AVE and factor loading are both above 0.5, so the model was confirmed to have excellent convergent validity.

Variable cross-loading, the Fornell–Larcker criterion, and the heterotrait–monotrait (HTMT) ratio are often used to evaluate discriminant validity [57]. In this study, the Fornell–Larcker criterion and HTMT ratio were chosen to test discriminant validity. For the Fornell–Larcker criterion, the $\sqrt{AVE}$ of each dimension should not be less than the correlation coefficient of the other dimensions [58] (Fornell and Larcker, 1981). As for the HTMT ratio, its value must not be greater than 0.85 [48]. Table 4 shows the discriminant validity testing results. Obviously, our model shows good discriminant validity under any test procedure.

#### 4.2.4. Structural Model

##### Goodness of Fit and Construct Correlation Analysis

Table 5 demonstrates the results for goodness of fit. We selected seven indicators to examine the fitness of the structural model and found that the values of all indicators reached the recommended level. Hence, it is appropriate to carry out structural modeling in this study.

A positive correlation was captured between HPR and PPA (*r* = 0.351, *p* < 0.05), HCO (*r* = 0.488, *p* < 0.01), and SNS (*r* = 0.475, *p* < 0.01); meanwhile, a positive consistency relationship between HPR and IFHB (*r* = 0.830, *p* < 0.01) was also confirmed (Table 6). The results provide transcendental support for hypothesis testing and structural modeling.

#### Predictive Ability

R^2^ and Q^2^ are two parameters that are commonly used to measure the applicability of PLS-SEM models. When the Q^2^ value calculated using the blindfolding method is greater than zero, the model is suitable for structural modeling; if R^2^ is above 0.5, it is considered to be acceptable. Table 7 shows the R^2^ and Q^2^ values of the model with and without consideration of the moderating effect of FSIR. Both of the models have sufficient predictive ability, and the latter is better than the former.

#### Path Analysis and Moderating Effect Results

**Figure 7:**
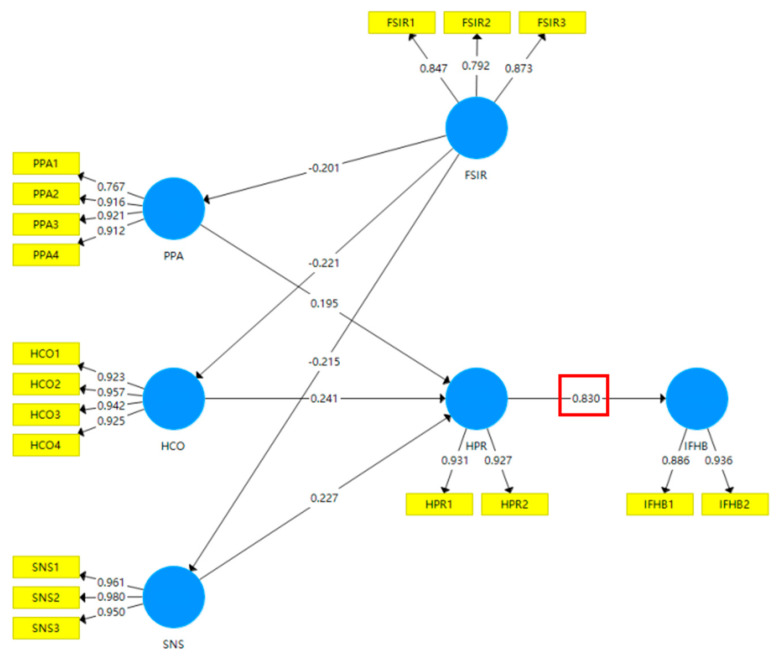
Model estimation results without consideration of the moderating effect of FSIR.

Figure 7 and Table 8 show the results of the model without considering the moderating effect of FSIR. HCO was the most important factor that revealed HPR (path coefficient, *PC* = 0.240; *p* < 0.05), followed by the impact of SNS (*PC* = 0.227; *p* < 0.05); the influence of PPA on HPR ranked last with a *PC* value of 0.195 (*p* < 0.01). The significant and positive effects of the above three factors on HPR fully support H1 and H2. These conclusions illustrate the fact that, during the pandemic, HPR has been largely shaped by external group influences rather than internal panic. FSIR had a negative effect on PPA, HCO, and SNS with *PC* values of −0.201 (*p* < 0.01), −0.221 (*p* < 0.01), and −0.215 (*p* < 0.01), respectively. Therefore, H4 was verified. This indicates that the establishment of an information release system helps to reduce the adverse effects of internal and external factors on citizens’ food hoarding preferences. Very importantly, HPR was shown to be a reliable indicator that forecasts IFHB with a *PC* value of 0.830 (*p* < 0.01), and thus H3 was verified. This means that blocking or limiting the conversion of HPR to IFHB may be a potential solution that can help control irrational food hoarding and reduce food waste.

**Figure 8:**
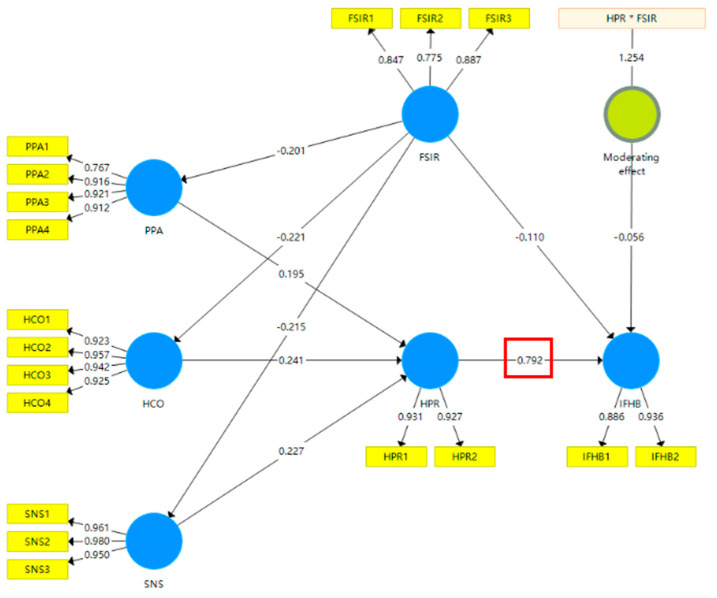
Estimation results of the moderating effect of FSIR.

Figure 8 shows the results of the model when the moderating effect of FSIR was considered. Table 9 presents the difference in coefficients for the “HPR -> IFHB” path with and without consideration of the moderating effect. It can be seen that, when FSIR was introduced into the model as a moderating factor, the path coefficient from HPR to IFHB decreased from 0.830 to 0.792 and the coefficient of the moderating effect was statistically significant (*p* < 0.05). This indicates that FSIR indeed played a significant and negative moderating role, thus validating H5.

#### Heterogeneity Analysis: The Impact of COVID-19 Exposure

The level of COVID-19 exposure, obviously, has an impact on food hoarding behavior. This is because, with different levels of exposure to the pandemic, citizens would have faced different food shortage risks, thus meaning there would have been differences in the perceived urgency of food hoarding. This has led to distinguished hoarding preferences and hoarding behaviors in reality. Citizens’ COVID-19 exposure was divided into two grades in this study: high exposure (*High_exposure*) and low exposure (*Low_exposure*). High exposure was defined as the occurrence of at least two sudden outbreaks of COVID-19 in the district (county) where the citizens lived in the past year, while low exposure was defined as no more than one sudden outbreak.

Table 10 presents the heterogeneity of the effects of PPA, HCO, SNS, and FSIR on HPR and IFHB under different levels of COVID-19 exposure. Welch–Satterthwaite test results showed that there were no significant differences in the effects of PPA, HCO, and SNS on HPR or the effects of HPR on IFHB between the two COVID-19 exposures, while there were significant differences in the effects of FSIR on PPA, HCO, and SNS and the moderating effect of FSIR. More precisely, compared to low-exposure areas, the release of food supply information in high-exposure areas had more obvious effects on reducing psychological panic, weakening group influence, and hindering the transformation of food hoarding preference into hoarding behavior.

### 4.2.5. Additional Insights: Influences of Control Variables

The ordinary least squares regression model (OLS) was used to reveal the influences of control variables on HPR and IFHB. The results of collinearity tests first verified that there was no strong multicollinearity among the explanatory variables. According to Table 11, age and household size had a positive impact on HPR at the statistical significance level of 5% and 10%, while professional training or a bachelor’s degree, a master’s degree and above, and the number of food purchases per week had a negative impact at a statistical significance level of 10%, 5%, and 10%, respectively. Except for professional training or a bachelor’s degree, the determinants of IFHB were exactly the same as those of HPR, although there were some differences in significance levels. The above results show that, first of all, older people are more likely to have food hoarding preferences and irrational food hoarding behavior. Secondly, the higher the education level of citizens, the weaker the food hoarding preference and the lower the probability of hoarding behavior. Thirdly, the larger the household, the less likely it is to reduce irrational food hoarding.

## 5. Discussions and Implications

Throughout the COVID-19 pandemic, moderate food hoarding has been used to not only meet people’s physical and safety needs, but also alleviate psychological panic and anxiety, which is conducive to individual self-protection. However, food products are highly perishable, and excessive hoarding will inevitably cause a serious food waste problem. At present, food supply at the production end is facing great challenges [59]. If food wastage cannot be prevented at the consumer end of the supply chain, it will be difficult for China to ensure food safety. During the pandemic, food waste caused by excessive food hoarding has accounted for the vast majority of total food waste in urban households. To reduce food waste, in addition to developing low-cost, innovative, eco-friendly approaches such as nanotechnology, using non-toxic, inexpensive, FDA-approved ingredients to guarantee the extension of food shelf-life while minimally affecting its organoleptic, nutritional, and sensory properties [60,61] becomes a necessary coping strategy to be used in conjunction with efforts to guide residents away from irrational hoarding behavior. Considering that previous studies have not revealed enough about the behavioral mechanism of citizens’ irrational food hoarding, it is necessary to carry out prospective exploration of this topic, and the relevant results will be of great value to the public policy design of food waste management.

This study was methodologically innovative as it involved the use of a more explanatory framework, the extended S-O-R, to explore Chinese citizens’ irrational food hoarding behavior during the COVID-19 pandemic. Measurement model results uncovered an excellent fit between the integrated model and the data, indicating that the research framework we developed is highly applicable in the specific context of the COVID-19 pandemic. Structural model results not only verified the positive consistency relationship between citizens’ food hoarding preference and hoarding behavior, but also revealed the main explanatory components of hoarding preference, including psychological panic, homology consistency, social network support, and hoarding preference itself. Furthermore, the moderating role of food supply information release in the transition from hoarding preference to hoarding behavior was also scientifically clarified. More importantly, hoarding preference occupied a large proportion of the variance in hoarding behavior, and psychological panic, homology consistency, and social network support explained a lot of the variance in hoarding preference. This indicates that the analytical framework established in this study was able to identify the main influencing factors of behaviors and preferences in this specific context as accurately as possible. In other words, the model has strong predictive power.

The most important finding of this study is that there was a positive correlation between food hoarding preference and irrational hoarding behavior. This result has a dual connotation. First, without external intervention, citizens’ high preference for food hoarding is eventually transformed into actual irrational hoarding behavior [62]. As described above, the irrational food hoarding behavior of citizens during the COVID-19 pandemic has resulted in huge food waste. Therefore, preventing the transformation of hoarding preference into hoarding behavior through external interventions emerges as a key component of food waste management [51]. This study found that government food supply information release helps to block the above transformation process. This means that by establishing a stable government food supply information release system and enhancing citizens’ understanding of food supply and security policies (such as stable supply channels and complete logistics chains, etc.), citizens’ perception of food availability and their trust in the government will be effectively improved. In this way, the irrational food hoarding behavior of citizens would be efficiently managed.

Another connotation is the possibility that irrational food hoarding behavior can be reduced by weakening food hoarding preference. As a matter of fact, previous studies have shown that changing individual preferences is an effective measure that can be used to regulate behavior [63]. So, what can be done to weaken the food hoarding preference of citizens in this study? From the perspective of the formation mechanism of hoarding preference, group influence (including homology consistency and social network support) occupied the largest proportion of the variance in hoarding preference, which suggests that weakening group influence is the most effective solution for decreasing food hoarding preference. Due to uncertainty regarding the impact of the pandemic on the food supply chain, it became difficult for people to rely on themselves to obtain effective information that they could use to support food hoarding decisions. At this point, citizens tend to use the behavior of others to guide their own behavior [64]. Under the influence of the group, individuals are more prone to impulsive behavior, which is the real cause of the phenomenon of “snapping up food” that has often observed during the pandemic.

The essence of group influence is herd mentality [65] (Paul, 2011). According to existing research, the herd mentality can be regulated by the following two measures. One is the enhancement of people’s self-control from the perspective of individuals [66], and the other is the cultivation of positive public opinion from the perspective of groups [67]. In this study, we accordingly proposed schemes to attenuate the group influence of food hoarding among citizens. On the one hand, diversified measures such as online and offline social education and informational hints in specific situations (posting slogans in shopping malls, etc.) should be taken to guide citizens’ food purchasing behavior toward a “return to rationality” [68]. Rational individuals are less susceptible to external interference. Of course, when they buy food, they will make the most reasonable decision based on their own demand preferences and actual demand, instead of blindly following trends. On the other hand, the chain of transmission for negative inducible information that exists between people (especially among acquaintances) should be interrupted [69]. The anxiety caused by false and negative information (rumors, etc.) related to the COVID-19 pandemic can cause people to lose their minds and become dominated by the crowd. Therefore, the government should take measures to strengthen controls on false and negative information in society and actively foster positive public opinion in relation to food consumption.

Alleviating psychological panic has also been found to play an important role in weakening food hoarding preferences. Throughout the COVID-19 pandemic, localized outbreaks have been capable of erupting at any moment. Nevertheless, citizens are unable to grasp the possible impact of potential outbreaks on the food supply chain. In other words, they are not sure whether their food needs could be guaranteed in the event of an outbreak. In this case, panic was triggered based on concerns about the availability of food. Panic comes from psychology [70]. Through necessary psychological intervention, citizens’ food hoarding preference can be effectively regulated, and household food waste problems can then be properly managed. Specifically, these psychological interventions can mainly be carried out from two aspects [71]. The first involves strengthening public dissemination of scientific information to improve citizens’ awareness of the regularity, harm, and coping strategies involved in the normalized prevention and control stage of COVID-19 so as to avoid psychological panic due to a lack of understanding of the pandemic itself. The second involves paying attention to the release of food supply information, improving citizens’ perception of food availability, and enhancing their satisfaction with the government’s food supply and guarantee efforts so as to eliminate panic and reduce non-essential food hoarding.

The role of food supply information release has been specifically emphasized in the above discussions. The release of food supply information can not only block the transformation of food hoarding preference into irrational food hoarding behavior, but also effectively alleviate the psychological panic of citizens and weaken the influence of groups. Furthermore, our study also found that the effect of food supply information release was better in areas with high pandemic exposure than in areas with low exposure. These findings provide support for regulating irrational food hoarding and reducing food waste by establishing a stable food supply information release mechanism. How the mechanism should work is important to consider. Ideally, an online information publishing platform should be set up to release price information and the daily availability of different foods in various local markets. Additionally, a food supply and guarantee press conference system should be established, that is, competent authorities should hold regular press conferences to publicize the local food production, procurement, logistics, inventory, and supply and demand situation, and the press conference should be broadcast simultaneously on different platforms. Through a variety of publicity channels, citizens should be exposed to relevant food information so that as many citizens as possible can accurately understand the status of local food supplies and have full confidence in the government’s food supply capacity.

The above findings provide some insights into how to solve the irrational food hoarding dilemma of citizens from the perspective of internal and external stimuli. It is worth noting that our extended study also found that citizens’ food hoarding preferences and hoarding behaviors can be also influenced by factors related to their individual and family characteristics. This influence can be described as the fact that highly educated and young people had weaker food hoarding preferences and were less likely to engage in hoarding behavior; however, larger family size had a negative effect on weakening hoarding preference and regulating hoarding behavior. These findings also have important implications for policy making. On the one hand, young people and highly educated groups should be the focus of attention; by encouraging them to reduce “impulsive shopping” and guiding them to establish a correct view of food consumption, it should be possible to reduce their unnecessary food hoarding to very low levels (Chen et al., 2019) [72]. On the other hand, Chinese urban households are generally small (more than 80% of households have no more than four people), which provides a rare opportunity to weaken food hoarding preference and regulate irrational hoarding behavior to the greatest extent; meanwhile, it also provides favorable external conditions for the sustainable management of food waste.

Actually, the contribution of this study goes beyond the particular case concerned here. For scholars and policy makers, it has broader implications. On the one hand, it is one of the few discussions currently exploring the issue of food waste caused by irrational hoarding during the COVID-19 pandemic, which is bound to provide novel insights into the development of sustainable food waste management policies. On the other hand, this study fully considered the realistic background of the pandemic when constructing its analytical model, and explanatory indicators included both individual psychological factors and collective action factors, which were the main inducements of citizens’ irrational decisions. Our research has confirmed that the model not only has high predictive accuracy, but also has strong explanatory power. The analytical model we constructed can be widely generalized, and the components, such as psychological panic and group influence, can also be introduced into other behavioral economics research conducted under specific scenarios.

## 6. Conclusions and Limitations

This study aimed to uncover the behavioral mechanisms underlying irrational food hoarding among the urban Chinese population during the COVID-19 pandemic. The following interesting findings were obtained. Firstly, 70.26% and 54.21% of citizens reported episodic and persistent food hoarding behavior, respectively. Among households with hoarding behavior, 66.37% had some degree of food waste. Secondly, food hoarding preference was the direct predictor of hoarding behavior, and there was a positive consistency relationship between them. This means that hoarding behavior can be effectively controlled by regulating preference. Thirdly, group influence (including homology consistency and social network support) and psychological panic enhanced citizens’ hoarding preference and induced hoarding behavior. Therefore, it is necessary to weaken group influence and try to help citizens overcome panic. Finally, government food supply information release can not only alleviate citizens’ psychological panic and weaken group influence, but also block the transformation of preference into behavior. Furthermore, information release works better in areas with high pandemic exposure than in areas with low exposure. These findings highlight the necessity of local governments establishing a stable food supply information release mechanism.

This study provides a novel understanding of the behavioral mechanisms underlying citizens’ irrational food hoarding during the COVID-19 pandemic. However, limitations inevitably exist. In this study, a limited number of cities were chosen for data collection, which limits the generalization of the results. Only three provincial capitals were selected in our study, while small and medium-sized cities were not included. In the future, more cities of different sizes should be selected to collect more representative samples for observation. Additionally, data were collected online in this study, which may raise the issue of selectivity bias. In fact, proper leading strategies should be used in the survey of measurement items, but online forms make it hard to achieve this. Therefore, face-to-face surveys should be conducted in the future to verify the reliability of online data.

## Figures and Tables

**Table 1:** Basic information of the respondents.

Variable	Description	Frequency	Ratio (%)
Gender	Male	333	65.17
	Female	178	34.83
Age	30 years old and below	196	38.35
	31–40 years old	186	36.40
	41–50 years old	91	17.81
	Over 50 years old	38	7.44
Highest education completed (Education1)	High school and below	39	7.63
(Education2)	Professional training or bachelor’s degree	261	51.08
(Education3)	Master’s degree and above	211	41.29
Marital status	1 = married	341	66.73
Household size	Less than 3	143	27.98
	3–4	268	52.45
	More than 4	100	19.57
Monthly household income	Less than CNY 10,000	115	22.50
	CNY 10,000–19,999	205	40.12
	CNY 20,000–29,999	80	15.66
	CNY 30,000 and above	111	21.72
Monthly household food expenditure	CNY 2000 and below	218	42.66
	CNY 2001–4000	173	33.86
	Over CNY 4000	120	23.48

Note: “CNY” refers to Chinese yuan, CNY 1 = USD 0.1480 (6 August 2022).

**Table 2:** Survey items related to citizens’ irrational food hoarding behavior.

Variable	Item	References
Psychological panic (PPA)		Boughton et al. [30]
PPA1	I often feel less well now compared to before the pandemic.	
PPA2	I often feel more nervous now compared to before the pandemic.	
PPA3	I often feel more anxious now compared to before the pandemic.	
PPA4	I often feel more upset now compared to before the pandemic.	
Homology consistency (HCO)		Raaij [49]
HCO1	When I see others hoarding food, I take it for granted that hoarding food is important.	
HCO2	When I see others hoarding food, I take it for granted that hoarding food is necessary.	
HCO3	When I see others hoarding food, I take it for granted that hoarding food is urgent.	
HCO4	When I see others hoarding food, I take it for granted that hoarding food is a wise choice.	
Social network support (SNS)		Li et al. [50]
SNS1	Relatives advised me to hoard food to address the potential risks of the pandemic.	
SNS2	Friends advised me to hoard food to address the potential risks of the pandemic.	
SNS3	Neighbors advised me to hoard food to address the potential risks of the pandemic.	
Hoarding preference (HPR)		Day et al. [51]; Becker et al. [52]
HPR1	I am more inclined to hoard food now than I was before the pandemic.	
HPR2	As long as the pandemic does not end, my food-hoarding tendencies will not abate.	
Irrational food hoarding behavior (IFHB)		Si et al. [53]; Wang et al. [54]
IFHB1	Episodic hoarding: I used to hoard food in large quantities during the pandemic.	
IFHB2	Persistent hoarding: since the pandemic, I have not stopped hoarding food in large quantities.	
Food supply information release (FSIR)		Li et al. [44]
FSIR1	The government’s food supply information release system is perfect.	
FSIR2	Government information release on food supply is always timely.	
FSIR3	Food supply information released by the government is reliable.	

**Table 3:** Reliability testing and convergent validity.

Item	Mean	S.D.	Factor Loading	Cronbach’s α	CR	AVE
PPA1	2.239	0.923	0.762	0.902	0.933	0.777
PPA2	2.695	1.058	0.916			
PPA3	2.767	1.104	0.923			
PPA4	2.734	1.044	0.915			
HCO1	3.387	0.896	0.923	0.954	0.966	0.878
HCO2	3.399	0.899	0.957			
HCO3	3.282	0.896	0.942			
HCO4	3.339	0.895	0.925			
SNS1	3.550	0.827	0.961	0.962	0.975	0.929
SNS2	3.501	0.823	0.980			
SNS3	3.448	0.826	0.950			
HPR1	3.386	0.985	0.931	0.842	0.927	0.863
HPR2	3.487	0.986	0.927			
FSIR1	3.740	0.800	0.845	0.787	0.874	0.699
FSIR2	3.732	0.857	0.753			
FSIR3	3.871	0.823	0.905			
IFHB1	3.450	1.008	0.886	0.799	0.907	0.830
IFHB2	3.855	0.939	0.936			

**Table 4:** Discriminant validity (Fornell–Larcker criterion and HTMT ratio).

Fornell–Larcker Criterion ^1^:						
	PPA	HCO	SNS	HPR	FSIR	IFHB
PPA	**0.882**					
HCO	0.357	**0.937**				
SNS	0.307	0.781	**0.964**			
HPR	0.351	0.488	0.475	**0.929**		
FSIR	−0.200	−0.220	−0.213	−0.312	**0.836**	
IFHB	0.352	0.441	0.466	0.830	−0.335	**0.911**
**HTMT ratio ^2^:**						
	PPA	HCO	SNS	HPR	FSIR	IFHB
PPA						
HCO	0.385					
SNS	0.329	0.816				
HPR	0.401	0.542	0.523			
FSIR	0.237	0.254	0.243	0.371		
IFHB	0.415	0.500	0.528	0.795	0.404	

Note: ^1^ The diagonal values (in bold) are the square root of the AVE values of the latent variables. ^2^ HTMT ratio < 0.85 is a threshold limit.

**Table 5:** Goodness of fit.

Index	Recommended Level	Estimate Value	GOF
χ^2^/df	<2.0	0.065	Yes
RMR	<0.05	0.037	Yes
RMSEA	<0.05	0.019	Yes
GFI	>0.9	0.932	Yes
AGFI	>0.9	0.985	Yes
NFI	>0.9	0.917	Yes
IFI	>0.9	0.964	Yes

**Table 6:** Construct correlations.

	PPA	HCO	SNS	HPR
HPR	0.351 **	0.488 ***	0.475 ***	
IFHB				0.830 ***

Note: ***^,^ ** significant at a 1% and 5% level, respectively.

**Table 7:** The coefficient of determination (R^2^) and cross-validated redundancy (Q^2^) values of the structural results.

	Without Moderating Effect		With Moderating Effect	
HPR	R^2^	0.490	R^2^	0.559
	Q^2^	0.212	Q^2^	0.243
IFHB	R^2^	0.688	R^2^	0.697
	Q^2^	0.155	Q^2^	0.290

**Table 8:** Detailed results of the structural equation.

Path	Estimate	*p*	Hypothesis	Supported
PPA -> HPR	0.195	***	H1	YES
HCO -> HPR	0.241	**	H2	YES
SNS -> HPR	0.227	**	H2	YES
FSIR -> PPA	−0.201	***	H4	YES
FSIR -> HCO	−0.221	***	H4	YES
FSIR -> SNS	−0.215	***	H4	YES
HPR -> IFHB	0.830	***	H3	YES

Note: ***, ** significant at a 1% and 5% level, respectively.

**Table 9:** Detailed results of the moderating effect of FSIR.

Path	Estimate	*p*	Hypothesis	Supported
Without moderating effect:				
HPR -> IFHB	0.830	***	H3	YES
With moderating effect:				
Moderating effect value	−0.056	**	H5	YES
HPR -> IFHB	0.792	***		

Note: ***, ** significant at a 1% and 5% level, respectively.

**Table 10:** Welch–Satterthwaite test for the effects of different COVID-19 exposure levels.

Path	Differences in Path Coefficients (*High_Exposure*-*Low_Exposure*)	t-Value |*High_Exposure* vs. *Low_Exposure*|	*p*-Value |*High_Exposure* vs. *Low_Exposure*|
PPA -> HPR	0.181	1.847	0.068
HCO -> HPR	0.091	0.441	0.660
SNS -> HPR	0.431	2.196	0.130
FSIR -> PPA	0.251	2.211	0.029
FSIR -> HCO	0.243	2.256	0.026
FSIR -> SNS	0.313	2.991	0.003
HPR -> IFHB	0.040	0.484	0.630
Moderating effect	0.027	0.245	0.007

**Table 11:** Influence of control variables on HPR and IFHB.

Variable	HPR	IFHB
Male	−0.001 (0.086)	−0.004 (0.085)
Age	0.006 ** (0.005)	0.003 ** (0.005)
Education		
High school and below	Omitted	Omitted
Professional training or bachelor’s degree	−0.098 * (0.085)	−0.095 (0.084)
Master’s degree and above	−0.157 ** (0.169)	−0.183 * (0.166)
Marital status	−0.280 (0.116)	−0.138 (0.114)
Household size	0.052 * (0.030)	0.019 ** (0.029)
Monthly household income	−0.002 (0.009)	−0.004 (0.009)
Monthly household food expenditure	0.101 (0.046)	0.082 (0.046)
Constant	3.671 *** (0.198)	3.759 *** (0.195)

Note: ***, **, * significant at a 1%, 5%, and 10% level, respectively. HPR and IFHB are weighted averages of their respective observable variables. “Marital status” is a dummy variable: 1 = married, 0 = otherwise. “Primary food shopper” is a binary variable: 1 = yes, 0 = no.

## Data Availability

The data presented in this study are available on request from the corresponding author.

## Abbreviations

IFHB, Irrational food hoarding behavior; PPA, Psychological panic; HCO, Homology consistency; SNS, Social network support; FSIR, Food supply information release; HPR, Hoarding preference; S-O-R theory, Stimulus-Organism-Response theory; PC, Path coefficient.
